# Good Clinical Practices for the Management of Post-Stroke Spasticity with BoNT-A: A Delphi-Based Approach from the Italian Expert Group

**DOI:** 10.3390/toxins18020094

**Published:** 2026-02-11

**Authors:** Alessio Baricich, Carmelo Chisari, Paolo De Blasiis, Marzia Millevolte, Alessandro Picelli, Andrea Santamato, Patrizia Maria Caglioni, Franco Molteni

**Affiliations:** 1 Department of Biomedical Sciences, Humanitas University, via Rita Levi Montalcini 4, Pieve Emanuele, 20072 Milan, Italy; 2 IRCCS Humanitas Research Hospital, via Manzoni 56, Rozzano, 20089 Milan, Italy; 3 Unit of Neurorehabilitation, Department of Neuroscience, University Hospital of Pisa, 56124 Pisa, Italy; 4 Department of Health Sciences, University of Basilicata, 85100 Potenza, Italy; 5 Neurorehabilitation Clinic, Marche University Hospital, 60126 Ancona, Italy; 6 Center for Research in Neuromotor and Cognitive Rehabilitation, Section of Physical Medicine and Rehabilitation, Department of Neuroscience, Biomedicine, and Movement, University of Verona, 37124 Verona, Italy; 7 Neurorehabilitation and Spinal Cord Rehabilitation and Functional Recovery Section, Policlinico di Foggia, 71122 Foggia, Italy; 8 Ipsen, 20124 Milan, Italy; patrizia.caglioni@ipsen.com; 9 Villa Beretta Rehabilitation Center, Valduce Hospital, Costa Masnaga, 23845 Lecco, Italy

**Keywords:** post-stroke spasticity, botulinum neurotoxin type A, good clinical practice, clinical management

## Abstract

Background: Post-stroke spasticity (PSS) is a common complication in stroke survivors, significantly impairing functional recovery and quality of life. Despite its prevalence, Italy lacks national guidelines or structured good clinical practice documents, resulting in heterogeneous clinical management. Methods: An Italian Delphi study was conducted to establish expert-based recommendations for PSS management. A panel of 93 rehabilitation medicine specialists and neurologists, each with over 5 years of experience in PSS management with botulinum toxin A (BoNT-A), participated in two rounds of voting on 47 statements drafted and approved by seven Key Opinion Leaders (KOLs), recognized for their national and international expertise. Consensus was defined as ≥75% of respondents answering ‘strongly agree’ or ‘somewhat agree’. Results: In Round 1, consensus was reached for 90% of statements; five items did not achieve the threshold. After revision and a second round, consensus was achieved for all items, including consideration of lesion site in clinical management and the role of adjuvant post-injection interventions. The panel’s heterogeneity ensured broad representativeness. Conclusion: This Delphi study provides the first structured Italian expert recommendations for PSS management. Full consensus was reached in all 47 statements and in the Symptoms domain, particularly regarding pain, stiffness and heaviness, which highlights the importance of a structured framework to support consistent, individualized care. By standardizing patient assessment, treatment planning, and follow-up strategies, these findings provide a practical reference for clinicians.

## 1. Introduction

### 1.1. Plain Language Summary

After a stroke, many people develop post-stroke spasticity (PSS), a condition that causes muscle stiffness and involuntary contractions, making movement and recovery difficult. Managing PSS can be challenging for doctors because symptoms and severity vary widely between individuals. In Italy, no national guidelines currently exist to help doctors treat PSS in a consistent way. To fill this gap, a group of Italian specialists carried out a Delphi consensus study, a structured process used to gather expert opinions. A total of 93 rehabilitation physicians and neurologists, all experienced in treating PSS with botulinum toxin type A (BoNT-A), reviewed and voted on 47 clinical statements covering six main areas of care: patient history, assessment, symptoms, treatment goals, BoNT-A therapy, and post-injection rehabilitation. During two rounds of voting, the experts reached agreement on all recommendations. The final guidance highlights the importance of (I) carefully assessing each patient’s history, cognitive function, and caregiving context; (II) tailoring BoNT-A injections using clinical evaluation and guiding tool such as ultrasound; (III) defining clear treatment goals and structured follow-up plans; (IV) using multimodal rehabilitation after injections, including stretching, positioning, electrostimulation, and new technologies like robotics and virtual reality. These expert-based recommendations provide the first structured Italian guidance for PSS management. They aim to help clinicians deliver consistent, individualized, and high-quality care, ultimately improving movement, independence, and quality of life for people living with post-stroke spasticity.

### 1.2. Stroke: Epidemiology and Risk Factors

Stroke remains one of the leading global health challenges, ranking as the second cause of death and the third cause of death and disability combined in adults, as measured by disability-adjusted life-years (DALYs) among non-communicable disorders. Globally, from 1990 to 2021, stroke cases rose significantly, with a 70% increase in incidence, 44% in deaths, and 86% in prevalence. Annual stroke incidence rates, standardized to the Italian population, range from 175 to 360 per 100,000 in men and from 130 to 273 per 100,000 in women [1]. Hypertension is the most prevalent risk factor in Italian stroke registries, affecting a majority of patients, followed by atrial fibrillation, hypercholesterolemia, diabetes, and smoking [2]. Metabolic risk factors account for 69% of all strokes, followed by environmental (37%) and behavioral (35%) risks [3].

### 1.3. Definition, Prevalence, and Current Academic Understanding of Post-Stroke Spasticity (PSS)

Post-stroke spasticity (PSS) is a common complication of stroke that results in impairments and limitations in the performance of activities. After stroke, PSS onset may range from right after the stroke up to 12 weeks. Its prevalence tends to peak around 4 weeks post-stroke, with most affected patients exhibiting signs within this timeframe [4]. Post-stroke spasticity develops in up to 40% of patients within 3–6 months following stroke, and around 20% of these individuals experience severe disability [5]. In stroke survivors, PSS poses a significant challenge and impact on the functional recovery and quality of life of affected individuals [6]. The negative impact of PSS affects both the physical and mental well-being of patients with stroke by limiting mobility, everyday activities, and social interactions [7]. Severe or disabling PSS affects approximately 15% of patients with stroke [8]. Despite its prevalence, the management of PSS remains challenging due to the heterogeneity in clinical presentation and response to therapy. From a pathophysiological point of view, PSS is considered a motor disorder characterized by a velocity-dependent increase in muscle tone and exaggerated reflexes arising from upper-motor-neuron lesions due to stroke-induced injury, and its incidence may vary depending on the time since the stroke event occurred [9,10]. As to the mechanism underlying the hyperexcitable stretch reflex in PSS, research suggests the involvement of supraspinal dysfunction as a possible origin, probably due to an imbalance between inhibitory and excitatory control of spinal stretch reflexes following cortical disinhibition after stroke [11,12]. This imbalance contributes to stretching reflex hyperexcitability, leading to muscle overactivity and exaggerated reflex responses to stimuli [6].

### 1.4. Post-Stroke Spasticity: Treatment and Management

PSS treatment encompasses different management strategies, including pharmacological treatment with systemic or focal botulinum toxin A (BoNT-A) and multimodal rehabilitation procedures such as physiotherapy, instrumental physical therapies and use of orthoses (e.g., limb casting) [7,10,13], with early management of PSS playing a crucial role [11,14]. Despite the availability of effective therapeutic strategies, there is substantial variability in clinical practice, which stems from differences in patient assessment, goal setting, treatment selection, and post-injection management. Several unmet clinical needs still characterize PSS management regarding the optimal timing of treatment, dosage selection, and the ideal time for follow-up [9]. A previous survey and expert opinion highlighted the importance of timely identification of PSS to provide optimal timing for treatment, since many patients receive their treatment with an average delay of 2 to 3 years after stroke [15]. Moreover, outpatients and community treatment appear limited and obsolete in terms of satisfying PSS long-lasting care and interventions [16].

### 1.5. Toward Standardized Good Clinical Practice for Post-Stroke Spasticity in Italy

To date, no national Italian guidelines or structured good clinical practice documents specifically dedicated to PSS management have been published. This lack of formal guidance was a key driver for the development of the present Delphi consensus. In our article, we present the findings of a Delphi consensus aimed at defining key principles that specifically address the assessment and management of post-stroke limb spasticity and related symptoms (e.g., pain and subjective discomfort) within a BoNT-A-based and multimodal rehabilitation framework; other post-stroke BoNT-A indications (e.g., dysphagia) were outside the scope of this Delphi. By incorporating the perspectives of experienced clinicians, this study seeks to standardize clinical decision-making, enhance treatment efficacy, and provide evidence-based guidance for optimizing patient outcomes. In addition to supporting clinical decision-making in complex cases, this work is intended to serve as a practical reference for clinicians, particularly those who are newly approaching the management of PSS or with limited prior experience, by offering expert-based recommendations across key domains of care. The results of this consensus process will contribute to a more structured and effective approach to PSS management, ultimately improving the standard of care for individuals affected by post-stroke motor impairments.

## 2. Results

The method and the composition of the scientific board committee and the panel of experts have been described in the Materials and Methods section. Round 1 (R1) was sent out on 9 January 2025, and finalized on 21 January 2025, with 93 members of the external panel voting on 47 statements. Participants were from northern (45%), central (21%), and southern (34%) Italy (Table 1) from hospital-based (81%) and university-affiliated (19%) settings; 34% were neurologists and 66% were physical medicine and rehabilitation specialists (Table 1).

All members were clinically experienced, with more than 5 years dedicated to the management and treatment of post-stroke spasticity (PSS) using botulinum toxin type A (BoNT-A) representing a broad range of clinical expertise and practice setting. The return rate was 100%. The list of statements submitted for the first e-Delphi round and their respective consensus are reported in Table 2.

Consensus was reached for almost 90% of the statements, while the following five statements did not achieve consensus: 1. The lesion site should influence clinical management; 43. In post-injection treatment, electrostimulation of the inoculated muscle is useful; 44. In post-injection treatment, the use of shock waves of the inoculated muscle is useful; 45. In relation to treatment goals, electrostimulation of antagonistic muscles is useful in post-injection treatment; 47. In relation to treatment goals, in post-injection treatment, the use of virtual reality is effective. Regarding the importance of medical history and multimodal post-inoculation treatment domains, only 25% and 50% of the statements reached consensus, respectively, while all the statements in the remaining domains reached consensus at R1. After R1, a new in-person meeting held by the scientific board was set up to discuss the results and revise the statements that did not reach consensus. The revised and amended statements for Round 2 (R2) are reported in Table 2. On 10 February 2025, R2 was sent out, and the results were collected on 23 February 2025. Seventy-three out of 93 panelists responded during R2 (return rate: 78.5%), and consensus was reached for all statements (Table 3).

## 3. Discussion

### 3.1. Interpretation of Consensus Findings

PSS is a common complication in stroke survivors, characterized by increased muscle tone and exaggerated reflexes, which can impair quality of life [17]. Given the high heterogeneity of symptoms and manifestations, PSS requires a standardized approach to act promptly and optimize clinical outcomes for patients [15,18]. By promoting a structured and shared clinical framework, these recommendations may also contribute to reducing delays in BoNT-A initiation, facilitating earlier identification of eligible patients and timely referral to treatment. The management of PSS involves several effective approaches, with BoNT-A considered the first-line treatment for focal PSS [19]. The aim of this Delphi study was to provide a list of good clinical practices for the management and treatment of on-limb PSS and its functional consequences, based on solid and broad consensus among Italian specialists. Most of the statements submitted to the extended panel during R1 achieved broad consensus, specifically reinforcing steps in patient evaluation, symptom assessment, and strategies for PSS treatment. The high level of agreement observed across most domains likely reflects a growing convergence in clinical practice, driven by increasing recognition of the complexity of PSS and the need for structured, yet flexible, management strategies.

### 3.2. Interpretation of Non-Consensus and Revised Statements

Although the first statement in the first domain achieved consensus only after revision and rephrasing during R2, the extended panel agreed on the importance of patient medical history. As reported in the literature, the site and size of the injury deeply influence the clinical management of PSS, as they may correlate with the severity of specific muscle groups [20,21]. Other studies have shown that lesions in certain cortical and sub-cortical regions are linked to upper-limb PSS, providing a strategic approach for this type of injury [22]. A more precise and efficient therapeutic approach may be achieved by analyzing different PSS profiles associated with specific lesion locations [23]. The type of lesion represents another crucial piece of information for the optimal management of PSS. Different types of strokes (ischemic or hemorrhagic) may lead to various patterns of muscle overactivity, necessitating different therapeutic approaches [21,24]. However, the initial lack of consensus likely reflected concerns about overly deterministic wording rather than disagreement on the clinical relevance of lesion characteristics. The revised statement, framed in a more inclusive and interpretative manner, facilitated agreement while preserving clinical applicability.

### 3.3. Clinical Implications

In addition to this revised statement, a high consensus was reached on the role of prior treatments (86.0%) and time since stroke (93.6%) in clinical decision-making, reinforcing evidence that these factors significantly impact PSS progression and therapeutic response (Table 2). The second domain addressed a multidimensional patient evaluation, integrating functional, cognitive, postural, and contextual aspects of care. Among these topics, panelists reached a consensus greater than 85% for all statements. The cognitive functional assessment represents a key intervention after stroke [25], and panelists agreed it should influence the clinical management of PSS. In this domain, the unanimous consensus on the importance of identifying a caregiver (100%) and the familial context underscores the well-recognized role of family and caregivers in optimizing rehabilitation outcomes [8]. Other statements in this domain showed a high level of consensus (ranging from 85% to 94%) on the segmental and postural assessment across different positions (supine, sitting, standing and sitting to standing), suggesting the importance of a comprehensive, multimodal evaluation approach in PSS, consistent with literature recommendations [26]. The uniformly high level of consensus in this domain underscores the shared recognition that PSS management extends beyond motor impairment, requiring consideration of cognitive status, caregiver support, and functional context to ensure treatment feasibility and adherence, as reported in the literature [27,28,29]. In the symptoms’ domain, six out of eight statements achieved full consensus after R1, while the timing of pain occurrence and the type of subjective disturbances, such as stiffness and heaviness, reached 98.9% consensus. Pain is common after stroke but often underrecognized [30]. The strong consensus for these statements highlights the importance of a comprehensive assessment of pain and subjective discomfort as routine evaluation of PSS. Similar results were observed in the therapeutic target domain. Experts strongly agreed on defining a primary treatment goal before initiating therapy (99.0%) and identifying at least one secondary goal (94.6%), in line with the literature [31]. Moreover, 100% consensus was achieved on the necessity of establishing a multimodal treatment before starting therapy. This complete agreement reflects the current recommendation for establishing a comprehensive, patient-specific, goal-centered, team-based rehabilitation program to achieve treatment goals [32,33]. Regarding the treatment with BoNT-A, a strong consensus was reached among all statements. The importance of instrumental guidance, such as ultrasound, electrical stimulation/electromyography, and ultrasonography guidance, during BoNT-A treatment for muscle localization and identification, has been acknowledged as fundamental by the panelists, confirming and supporting literature on the matter [21,34]. As reported in the literature, instrumental guidance can improve accuracy of injections, although there is no evidence to support one technique over the others [35]. Moreover, dynamic electromyography (89.2%) and diagnostic motor nerve block (88.1%) were considered useful, in case of clinical uncertainty, for functional confirmation when selecting target muscles. Moreover, dosage modulation and re-injection timing were crucial for panelists, who agreed that individual muscle dosing should be guided by clinical evaluation and decisions regarding reinoculation after clinical reassessment and the establishment of new treatment goals. They also agreed that reinoculation should not occur sooner than three months after the previous injection, consistent with practice recommendations [36]. Finally, panelists expressed agreement on the importance of using validated clinical scales, such as International Classification of Functioning, Disability and Health (ICF), for functional assessment (84.9%), emphasizing standardized outcome measures. The use of standardized clinical scales and predefined follow-up timing may improve the consistency and comparability of patient monitoring over time, supporting a more uniform and structured follow-up across different clinical settings. In the final domain, the multimodal inoculation treatment domain, half of the statements achieved consensus only after rephrasing, suggesting that more inclusive and flexible wording was needed. The consensus reached during R2 highlighted the importance of electrostimulation of the injected and antagonist muscles, the use of shock waves, virtual reality, robotic technologies, and a structured stretching program and positioning techniques in post-injection treatment. The need for rephrasing in this domain likely reflects heterogeneity in access to resources and variability in clinical practice rather than disagreement on the conceptual value of adjunctive therapies. More flexible wording allowed panelists to endorse these interventions as optional, goal-oriented components of a multimodal strategy. Although the response rate decreased in Round 2, it remained within acceptable ranges for Delphi studies and involved the same expert panel. Given the focused nature of the second round and the high level of agreement achieved, this reduction is unlikely to have substantially affected the robustness or representativeness of the final recommendations.

### 3.4. Limitations of the Study

This study presents limitations that should be acknowledged. First, the consensus was developed within a national Italian context, which may limit the generalizability of the findings to other healthcare systems with different organizational structures, resources, and clinical pathways. Second, although the recommendations place strong emphasis on patient-centered care, patient or caregiver representatives were not directly involved in the Delphi process, and their perspectives were therefore not explicitly captured. Third, as with all Delphi-based studies, the recommendations rely on expert opinion rather than primary empirical data. While this approach is valuable in areas where evidence is limited or heterogeneous, the resulting statements should be interpreted as expert-informed guidance rather than high-level evidence-based recommendations. Nonetheless, the structured Delphi methodology, anonymous voting, and predefined consensus thresholds were designed to enhance methodological rigor and reduce individual bias. In addition, the Delphi panel did not formally include other healthcare professionals involved in spasticity management, such as rehabilitation nurses or neuroelectrophysiology specialists, whose perspectives may further enrich future multidisciplinary consensus efforts. Moreover, safety considerations for BoNT-A, including assessment of prior treatment reactions and monitoring for adverse effects such as muscle weakness, were not addressed, since they are detailed in the product summary of characteristics and national/international guidelines; readers are referred to these for comprehensive risk management.

## 4. Conclusions

This Delphi consensus study established expert-driven recommendations for assessing and managing PSS, focusing on six key domains: medical history, patient evaluation, symptom assessment, therapeutic targets, BoNT-A treatment, and multimodal post-injection rehabilitation. It represents the first Italian collection of structured good clinical practice framework for the management of PSS. The high level of agreement among most statements reflects a strong alignment among Italian experts regarding the importance of structured and individualized approaches in PSS management. Full consensus in the symptom’s domain, particularly on pain and other subjective disturbances such as stiffness and heaviness, highlights the importance of a standardized framework to support consistent, individualized care and underscores the need for routine evaluation of patient discomfort to optimize clinical outcomes and guide structured assessment, treatment planning, and follow-up. Initial areas of non-consensus in domains 1 and 6 were resolved after statement revisions and a second round of voting, demonstrating the value of iterative refinement in achieving clearer, more clinically applicable recommendations. The amended statements underscored the significance of considering the site of the lesion in clinical decision-making and refined the role of post-injection interventions (e.g., electrostimulation, shock waves, and innovative technologies in rehabilitation strategies). These adjustments align with current evidence on neurorehabilitation and emerging adjunctive therapies, emphasizing the need for further research to optimize these approaches. This Delphi consensus was developed to address this need by providing expert-based recommendations across the most relevant domains of care. This work contributes to the existing literature by providing one of the first structured, expert-based consensus documents focused on the clinical management of PSS among Italian clinicians. It addresses areas not yet covered by national guidelines, offering practical, experience-driven recommendations across key domains of care. As the field progresses, it is crucial that research, policy, and clinical practice remain aligned toward truly patient-centered care. In this context, the consensus may be particularly valuable for less specialized or lower-volume centers, providing clear guidance to support clinical decision-making, reducing variability in practice, and promoting more homogeneous standards of care. Embracing a multidimensional view of health that integrates biological, psychological, and environmental factors may enhance the understanding and management of neurological impairments. Extending this comprehensive approach beyond neurorehabilitation could promote more personalized and effective care across medical disciplines [37]. Overall, these findings reinforce the importance of a comprehensive, multimodal approach to PSS treatment, integrating precise patient assessment, goal-directed therapy, and evidence-based interventions to improve clinical outcomes.

## 5. Materials and Methods

This study employed the Delphi method to achieve expert consensus on good clinical practice for the management of PSS. An independent service provider ensured participant anonymity and minimized potential biases, further enhancing the validity and reliability of the consensus. The Delphi method is a structured process designed to facilitate expert discussions on complex issues [38,39], and is widely applied across various fields, particularly in healthcare (where it helps establish consensus in areas lacking robust data, comprehensive guidelines, or well-defined knowledge) [40,41]. To be effective, the Delphi method follows three key stages: selecting a panel of experts based on their recognized international and/or national clinical expertise, significant scientific contributions through publications and research, active involvement in professional societies, and experience in peer-reviewed work; efforts were made to ensure a balanced representation of professionals with diverse perspectives within the field; developing structured surveys; and conducting iterative rounds to refine responses and reach consensus [41]. The iterative nature of this process aims to progressively narrow responses while ensuring consensus through three fundamental principles: anonymity, controlled feedback, and statistical aggregation of group responses [36]. This Delphi consensus study was designed and conducted between January 2025 and March 2025. A scientific board of 7 Italian Key Opinion Leaders in rehabilitative medicine was established based on their clinical expertise in PSS management. The Key Opinion Leaders were selected for their established academic and clinical expertise in post-stroke spasticity and botulinum toxin type A treatment. Board members were nationally and internationally recognized experts with extensive experience in clinical practice, research, and scientific dissemination in the field. The Scientific Board was responsible for defining the study domains, overseeing statement development, and ensuring adherence to Delphi methodology. The initial set of statements for the first-round Delphi questionnaire was developed through a structured, multistep process. First, three in-person advisory board meetings were organized, involving senior neurologists and rehabilitation medicine specialists with extensive clinical experience in the management of post-stroke spasticity. During these meetings, participants were engaged in structured discussions aimed at mapping the entire clinical pathway of post-stroke limb spasticity management (upper and/or lower limb), from patient assessment and symptom evaluation to therapeutic goal setting, botulinum toxin type A treatment, and post-injection management. The six key domains (medical history, patient evaluation, symptom assessment, therapeutic targets, BoNT-A treatment, and multimodal post-injection rehabilitation) were identified and refined by the Steering Committee, based on their collective expertise. These domains formed the basis for developing the initial statements. Key clinical actions and decision points considered essential for good clinical practice were identified within each domain. These elements were subsequently translated into preliminary statements, ensuring neutral and unambiguous wording suitable for Delphi voting. The scientific board then reviewed, refined, and consolidated the preliminary items to eliminate redundancies and ensure consistency with Delphi methodology principles, resulting in a final set of 47 statements submitted for Round 1 of the Delphi process. The definitive 47 statements were defined for the first-round questionnaire, which was distributed via an electronic platform to a panel of 93 Italian expert rehabilitation medicine specialists with expertise in PSS. The Italian version of the 47 statements approved and amended is reported in Table S1 and Table S2, respectively. The 93 Italian neurologists and rehabilitation medicine specialists were selected based on their recognized clinical expertise and experience in the management of post-stroke spasticity, including the use of botulinum toxin type A. Selection was based on professional competence and direct involvement in routine clinical care rather than on predefined criteria related to clinical setting or institutional affiliation. This panel was invited to vote on each statement to gather consensus according to the Delphi methodology. The level of agreement or disagreement on each statement was expressed individually and anonymously using a five-point Likert-type scale (1 = strongly disagree, 2 = somewhat disagree, 3 = neither agree nor disagree, 4 = somewhat agree, and 5 = strongly agree). During Round 1 (R1), if panelists indicated a level of agreement lower than 5/5, they were required to explain their disagreement in a comment. Comments were carefully reviewed and integrated to refine the questionnaire for the second round (R2) of the analysis. Consensus for each statement was deemed achieved when ≥75% of respondents answered either ‘strongly agree’ or ‘somewhat agree’. This threshold was chosen a priori based on methodological precedent in Delphi research, where a ≥75% agreement has been shown to represent a commonly used and median level for defining strong expert consensus across health-related Delphi studies [42,43]. Subsequently, the expert board finalized the R2 questionnaire, which included a revised version of the statements that did not reach consensus in R1. During R2, the 93 panelists were then invited to evaluate and vote on the statements included in the revised questionnaire. A total of 73 out of 93 experts voted in Round 2. The final stage involved comprehensive data analysis and the development of the final Delphi report, with guidance from a methodology expert.

## Figures and Tables

**Table 1:** Geographical distribution and specializations of the expert panel.

Panelists’ Specialization	%
Neurologist	34
Rehabilitation medicine specialists	66
**Panelists’ gender**	**%**
Female	48
Male	52
**Panelists’ geographical distribution**	**%**
Northern Italy	45
Central Italy	21
Southern Italy	34

**Table 2:** Statements submitted for the first e-Delphi round (R1). Grey rows include statements that did not reach consensus.

Domain: Importance of Medical History	Consensus (%)
The lesion site should influence clinical management.	73.1%
2. The type of lesion should influence clinical management.	76.4%
3. The time elapsed since the stroke event should influence clinical management.	93.6%
4. Previous treatments should be considered when determining clinical management.	86.0%
**Domain: Patients’ evaluation**	
5. The assessment of cognitive function should influence clinical management.	89.3%
6. The patient’s familial and caregiving context should influence clinical management.	87.1%
7. Whenever possible, a caregiver should be identified.	100%
8. The patient’s segmental assessment should be performed in the positions deemed most useful by the clinician (supine and/or seated and/or standing).	89.3%
9. The patient’s postural assessment should be performed in the positions deemed most useful by the clinician (supine and/or sitting and/or standing).	88.2%
10. The assessment of associated postural reactions should be performed during the transition from sitting to standing.	88.2%
11. The assessment of associated postural reactions should be performed during ambulation.	94.7%
12. The assessment of the static postural pattern should be performed in the standing position.	92.5%
13. The assessment of the static postural pattern should be performed in the sitting position.	85.0%
14. Functional assessment of the upper limb movements, including reaching, grasping, and pinching, should be performed in a seated position.	94.6%
**Domain: Symptoms**	
15. During symptom assessment, the location of pain should be evaluated.	100%
16. During symptom assessment, the time of day when pain is perceived (nocturnal/diurnal) should be evaluated.	98.9%
17. During symptom assessment, pain perceived at rest should be evaluated.	100%
18. During symptom assessment, pain perceived during passive mobilization should be evaluated.	100%
19. During symptom assessment, pain perceived during active mobilization should be evaluated.	100%
20. During symptom assessment, the presence of subjective discomfort (such as stiffness and/or heaviness) potentially related to PSS should be evaluated	98.9%
21. During symptom assessment, the type of subjective disorder (such as stiffness and/or heaviness) potentially related to PSS should be evaluated.	100%
22. During symptom assessment, the site of subjective disturbance (such as stiffness and/or heaviness) potentially related to PSS should be evaluated.	100%
**Domain: Therapeutic target**	
23. Before determining the treatment, a primary goal should be defined.	99.0%
24. Before determining the treatment, at least one secondary goal should be identified.	94.6%
25. Before initiating treatment, a multimodal treatment plan should be established.	100%
**Domain: Treatment with BoNT-A**	
26. Regarding BoNT-A treatment, it is recommended to identify target muscles using at least one instrumental method (ultrasound, electromyography, or electrical stimulation).	96.8%
27. In case of clinical uncertainty, muscle localization using ultrasound alone is insufficient for BoNT-A treatment, but dynamic electromyography is recommended to functionally define the target muscle.	89.2%
28. In case of clinical uncertainty for BoNT-A treatment, it is useful to identify the target muscle with diagnostic anesthetic blockade.	88.1%
29. Regarding BoNT-A treatment, the appropriate dosage for each individual muscle should be determined through clinical assessment.	96.8%
30. Regarding BoNT-A treatment, it is useful to calibrate the appropriate dosage for each muscle based on its morpho-structural characteristics as assessed by ultrasound.	89.2%
31. The possibility of variable dilution should be considered in relation to the total dosage administered during a single treatment session.	77.4%
32. Regarding BoNT-A treatment, the timing of re-injection should be based on clinical evaluation.	95.7%
33. Regarding BoNT-A treatment, it is necessary for the multimodal treatment plan to be correlated with comprehensive clinical evaluation.	100%
34. Follow-up should be planned at the time of BoNT-A treatment administration.	97.9%
35. For BoNT-A treatment, it is recommended to assess treatment efficacy between 4 and 6 weeks after the first injection.	94.6%
36. Re-injection of BoNT-A must only be performed after a complete clinical reassessment.	96.8%
37. It is recommended that re-injection of BoNT-A occurs after a new definition of treatment goals.	89.3%
38. It is recommended that BoNT-A re-injection should not occur three months before the previous injection.	92.5%
39. Regarding BoNT-A treatment, all clinical and functional assessments must be conducted using validated clinical scales for evaluating “human functioning”, encompassing body structure, body function, activity, and social participation (ICF framework).	84.9%
**Domain: Multimodal post inoculation treatment**	
40. Post-injection treatment should be planned in accordance with the treatment goals.	98.9%
41. In relation to treatment goals, a structured stretching program should be defined as part of the post-injection management.	94.6%
42. In post-injection treatment, the use of positioning techniques such as casting, splints, orthoses, and taping should be considered.	97.9%
43. In post-injection treatment, electrostimulation of the inoculated muscle is useful.	69.9%
44. In post-injection treatment, the use of shock waves of the inoculated muscle is useful.	49.4%
45. In relation to treatment goals, electrostimulation of antagonistic muscles is useful in post-injection treatment.	57.0%
46. In relation to treatment goals, the use of robotic technologies can be useful in post-injection rehabilitation.	79.5%
47. In relation to treatment goals, in post-injection treatment, the use of virtual reality is useful.	71%

**Abbreviations**: BoNT-A, Botulinum Neurotoxin type A; ICF, International Classification of Functioning, Disability and Health.

**Table 3:** Amended statements voted during the second e-Delphi round (R2).

Amended Statements	Consensus (%)
**1.** Lesion site should be considered when defining clinical management.	87.5%
**43.** In post-injection treatment, electrostimulation of the inoculated muscle may be useful to facilitate internalization of the BoNT-A.	86.1%
**44.** In post-injection treatment, the use of shock waves in the inoculated muscle may be useful as an adjuvant treatment.	76.4%
**45.** In post-injection treatment, to achieve goals of active function, electrostimulation of antagonistic muscles may be useful.	88.9%
**47.** In relation to treatment goals, in post-injection treatment, the use of virtual reality is useful.	93.0%

**Abbreviations:** BoNT-A, Botulinum Neurotoxin type A.

## Data Availability

Qualified researchers may request access to the anonymized data underlying the findings reported in this publication by contacting an Ipsen representative. Additional relevant documents, such as the study protocol and analysis plan, may also be available upon request. Further information on Ipsen’s Data Sharing policy is available here (https://www.ipsen.com/science/clinical-trials/clinical-data-transparency/, accessed on 26 January 2026).

## Supplementary Materials

The following supporting information can be downloaded at: https://www.mdpi.com/article/10.3390/toxins18020094/s1, Table S1: Original Italian statements submitted for the first e-Delphi round (R1). Grey rows include statements that did not reach consensus; Table S2: Original Italian amended statements included in the second e-Delphi round (R2) of voting.

## Abbreviations

The following abbreviations are used in this manuscript:

BoNT-A	Botulinum Neurotoxin type A
ICF	International Classification of Functioning, Disability and Health
PSS	Post-stroke Spasticity
