# Supplementary material for: Good Clinical Practices for the Management of Post-Stroke Spasticity with BoNT-A: A Delphi-Based Approach from the Italian Expert Group
Source: Toxins (Basel). 2026 Feb 11;18(2):94. doi: 10.3390/toxins18020094 (PMC12945191; doi:10.3390/toxins18020094)
Supplement: Supplementary file 1 [file toxins-18-00094-s001.zip › toxins-4078071-supplementary.pdf]

Table S1. Original Italian statements submitted for the first e-Delphi round (R1). Grey rows include statements that did not reach consensus.

**Domain: Rilevanza dell'anamnesi**
1. La sede di lesione deve influenzare il management clinico
2. Il tipo di lesione deve influenzare il management clinico
3. Il tempo trascorso dall'evento deve influenzare il management clinico
4. I trattamenti precedenti devono influenzare il management clinico
**Domain: Valutazione del paziente**
5. L'inquadramento dell'ambito cognitivo deve influenzare il management clinico
6. La condizione famigliare – assistenziale del paziente dovrebbe influenzare il management clinico
7. Laddove possibile, è bene identificare un caregiver
8. La valutazione segmentale del paziente deve essere effettuata nelle posizioni considerate più utili dal clinico (supino e/o seduto e/o eretto)
9. La valutazione posturale del paziente deve essere effettuata nelle posizioni considerate più utili dal clinico (supino e/o seduto e/o eretto)
10. La valutazione delle reazioni posturali associate deve essere effettuata nei passaggi seduto-eretto
11. La valutazione delle reazioni posturali associate deve essere effettuata durante la deambulazione
12. La valutazione del pattern posturale statico deve essere effettuata in posizione eretta
13. La valutazione del pattern posturale statico deve essere effettuata in posizione seduta
14. La valutazione del gesto funzionale dell'arto superiore di reaching, grasping e pinch deve essere effettuata da seduto
**Domain: Sintomi**
15. Al momento della valutazione dei sintomi, deve essere valutata la sede del dolore
16. Al momento della valutazione dei sintomi, deve essere valutato il periodo della giornata durante il quale il dolore viene percepito (notturno/diurno)
17. Al momento della valutazione dei sintomi, deve essere valutato il dolore percepito a riposo
18. Al momento della valutazione dei sintomi, deve essere valutato il dolore percepito durante mobilizzazione passiva
19. Al momento della valutazione dei sintomi, deve essere valutato il dolore percepito durante mobilizzazione attiva

20. Al momento della valutazione dei sintomi, deve essere valutata la presenza del disturbo soggettivo (senso di rigidità e/o di pesantezza) correlabile alla spasticità
21. Al momento della valutazione dei sintomi, deve essere valutata la tipologia del disturbo soggettivo (senso di rigidità e/o di pesantezza) correlabile alla spasticità
22. Al momento della valutazione dei sintomi, deve essere valutata la sede del disturbo soggettivo (senso di rigidità e/o di pesantezza) correlabile alla spasticità
**Domain: Definizione obiettivi**
23. Prima di decidere il trattamento è necessario definire un goal primario
24. Prima di decidere il trattamento è bene identificare almeno un goal secondario
25. Prima del trattamento deve essere definito il piano multimodale di trattamento
**Domain: Trattamento con tossina botulinica**
26. In relazione al trattamento con tossina botulinica, è raccomandabile identificare i muscoli target con almeno un supporto strumentale (US, elettromiografia ed elettrostimolazione)
27. In caso di dubbio clinico, per il trattamento con tossina botulinica non è sufficiente la localizzazione del muscolo target con la sola valutazione ecografica, ma è raccomandabile ricorrere anche all'elettromiografia dinamica per definire il muscolo target da un punto di vista funzionale
28. In caso di dubbio, per il trattamento con tossina botulinica è utile identificare il muscolo target con blocco anestetico diagnostico
29. In relazione al trattamento con tossina botulinica, è necessario che il dosaggio appropriato per singolo muscolo venga identificato tramite valutazione clinica
30. In relazione al trattamento con tossina botulinica, è utile che il dosaggio appropriato per singolo muscolo sia calibrato in base alle condizioni morfologiche morfostrutturali del muscolo valutate con ecografia
31. Si deve considerare la possibilità di una diluizione variabile in rapporto ai dosaggi totali della singola seduta di trattamento
32. In relazione al trattamento con tossina botulinica, è necessario che timing di re-inoculazione avvenga sulla base della valutazione clinica
33. In relazione al trattamento con tossina botulinica, è necessario che il piano di trattamento multimodale venga correlato a valutazione clinica globale
34. È necessario che il follow-up venga pianificato al momento dell'effettuazione del trattamento con tossina botulinica
35. In relazione al trattamento con tossina botulinica, è raccomandabile valutare l'efficacia del trattamento tra le 4 e le 6 settimane dopo la prima inoculazione
36. È necessario che la re-inoculazione di tossina botulinica avvenga dopo una completa rivalutazione clinica
37. È raccomandabile che la re-inoculazione di tossina botulinica avvenga dopo una ri-definizione dei goal di trattamento

38. È raccomandabile che la re-inoculazione di tossina botulinica avvenga non prima dei 3 mesi dalla precedente inoculazione
39. In relazione al trattamento con tossina botulinica, tutte le valutazioni cliniche e funzionali devono essere effettuate in base a scale cliniche validate per la valutazione dello "human functioning" nelle sue componenti di struttura corporea, funzione corporea, attività e partecipazioni sociali (ICF)
**Domain: Trattamento post inoculo multimodale**
40. È necessario che il trattamento post inoculo sia pianificato in relazione ai goal del trattamento
41. In relazione ai goal di trattamento, nel trattamento post inoculo è necessario definire un programma di stretching
42. Nel trattamento post inoculo, è utile considerare l'utilizzo di tecniche di posizionamento come casting, tutori, ortesi e taping
43. Nel trattamento post inoculo, è utile l'elettrostimolazione del muscolo inoculato
44. Nel trattamento post inoculo, è utile l'utilizzo di onde d'urto del muscolo inoculato
45. In relazione ai goal di trattamento, nel trattamento post inoculo è utile l'elettrostimolazione dei muscoli antagonisti
46. In relazione ai goal di trattamento, nel trattamento post inoculo è utile l'utilizzo di tecnologie robotiche
47. In relazione ai goal di trattamento, nel trattamento post inoculo è utile l'utilizzo della realtà virtuale

Table S2. Original Italian amended statements included in the second e-Delphi round (R2) of voting.

1. La sede di lesione deve essere presa in considerazione nella definizione del management clinico
43. Nel trattamento post inoculo, l'elettrostimolazione del muscolo inoculato può essere utile per facilitare l'internalizzazione della tossina
44. Nel trattamento post inoculo, l'utilizzo delle onde d'urto nel muscolo inoculato può essere utile come trattamento adiuvante
45. Nel trattamento post inoculo, per il raggiungimento di goal di funzione attiva, può essere utile l'elettrostimolazione dei muscoli antagonisti
47. In relazione ai goal di trattamento, nel trattamento post inoculo è utile l'utilizzo della realtà virtuale
